# Diabetes Impairs the Virological Response in Patients with Chronic Hepatitis B: Glycemic Control as a Key Modifiable Risk Factor

**DOI:** 10.3390/jcm15051826

**Published:** 2026-02-27

**Authors:** Aoyi Li, Yan Han, Guanglin Xiao, Zhiling Deng, Chaojing Wen, Ke Qiu, Taiyu He, Hong Ren

**Affiliations:** Key Laboratory of Molecular Biology for Infectious Diseases (Ministry of Education), Department of Infectious Diseases, Institute for Viral Hepatitis, The Second Affiliated Hospital, Chongqing Medical University, Chongqing 401336, China; leeayc@icloud.com (A.L.);

**Keywords:** chronic hepatitis B, type 2 diabetes mellitus, virological response, glycemic control

## Abstract

**Background/Objectives**: Chronic hepatitis B (CHB) and type 2 diabetes mellitus (T2DM) frequently coexist. This study aimed to investigate the impact of T2DM and glycemic control on antiviral efficacy in CHB patients. **Methods**: This single-center, retrospective cohort study included treatment-naïve CHB patients who initiated nucleos(t)ide analogue (NA) therapy between January 2019 and January 2024. The primary endpoint was a complete virological response (CVR), defined as achieving HBV DNA levels below 20 IU/mL after 48 weeks of treatment. **Results**: The CHB + T2DM group (*n* = 81) demonstrated a significantly lower CVR rate than the CHB group (*n* = 106) (26.0% vs. 41.2%, *p* = 0.038). Multivariate analysis identified T2DM as an independent negative predictor of a CVR (*OR* = 0.400, 95% *CI:* 0.196–0.815, *p* = 0.012). Within the CHB + T2DM subgroup, adequate glycemic control (HbA1c < 7%) was associated with a higher CVR (38.7% vs. 16.7%, *p* = 0.034). Patients newly diagnosed with diabetes at enrollment showed a higher rate of HBeAg loss than those with pre-existing diabetes (57.1% vs. 10.0%, *p* = 0.036). Regarding antiviral regimens, entecavir-treated CHB + T2DM patients had a lower CVR than CHB controls (18.8% vs. 46.2%, *p* = 0.015). Furthermore, tenofovir-based regimens showed a more favorable antiviral trend than entecavir in CHB patients with T2DM. **Conclusions**: Comorbid T2DM was an independent risk factor for impaired antiviral efficacy in CHB patients. Optimal glycemic control may improve virological outcomes. These findings suggest that the early diagnosis and management of T2DM could enhance antiviral treatment efficacy in CHB patients.

## 1. Introduction

Chronic hepatitis B (CHB) virus infection remains a significant global public health challenge, affecting an estimated 296 million people worldwide and conferring a markedly elevated risk of life-threatening complications, including liver cirrhosis, hepatic decompensation, and hepatocellular carcinoma (HCC) [1,2]. The cornerstone of CHB management is long-term antiviral therapy with first-line agents such as entecavir, tenofovir, or pegylated interferon [3,4]. The primary goal of antiviral therapy is to achieve sustained viral suppression, defined as the durable maintenance of undetectable HBV DNA levels in the blood, which is essential for halting disease progression and reducing liver-related morbidity and mortality [5].

According to current guidelines, therapeutic endpoints for CHB include the biochemical response (alanine aminotransferase [ALT] normalization), virological response (undetectable hepatitis B virus [HBV] DNA), serological response (hepatitis B e antigen [HBeAg] loss or seroconversion), functional cure (hepatitis B surface antigen [HBsAg] loss), and complete cure (covalently closed circular DNA [cccDNA] clearance) [6,7,8]. While most CHB patients achieve viral suppression with first-line nucleos(t)ide analogues (NAs), a considerable number still show suboptimal or delayed responses [9,10,11,12]. Existing studies have confirmed that the HBV DNA load exhibits a continuous, graded positive correlation with the incidence of HCC and serves as an independent predictor of cirrhosis development [13,14,15]. Achieving sustained suppression of HBV DNA can reduce the risk of HCC and reverse liver fibrosis and cirrhosis [14,16]. Identifying high-risk individuals and clarifying modifiable factors are therefore essential for optimizing personalized treatment strategies [17].

Type 2 diabetes mellitus (T2DM) is a frequent comorbidity in patients with chronic hepatitis B (CHB) and contributes to poorer hepatic outcomes [18,19]. The evidence suggests that CHB is associated with increased risks of insulin resistance and T2DM. A meta-analysis reported a 1.33-fold higher risk of T2DM in HBV-infected individuals than in uninfected controls (95% CI: 1.09–1.62) [20]. A cross-sectional study of 7880 Korean adults also found that CHB was independently associated with greater insulin resistance [21]. Cohort data from Taiwan showed that CHB patients who developed diabetes had elevated risks of liver cirrhosis (aHR: 2.02), hepatic decompensation (aHR: 1.79) [22], and hepatocellular carcinoma (HCC; aHR: 1.8) [23]. In summary, numerous studies indicate that T2DM can exacerbate adverse liver outcomes in CHB patients. Although prior research identifies antiviral therapy as a significant independent predictor of HCC in this comorbid population [24], clear clinical evidence showing how T2DM specifically affects the efficacy of antiviral treatment is still lacking.

Therefore, we conducted a retrospective study to comprehensively compare virological and biochemical response rates in treatment-naïve patients with and without diabetes after 48 weeks of antiviral treatment. Additionally, we examined the relationship between glycemic control (HbA1c levels) and treatment efficacy in diabetic patients.

## 2. Materials and Methods

### 2.1. Study Population

This was a single-center, retrospective cohort study. We enrolled treatment-naïve adult patients with chronic hepatitis B (CHB) who initiated antiviral therapy with entecavir (ETV), tenofovir disoproxil fumarate (TDF), tenofovir alafenamide (TAF), or tenofovir amibufenamide (TMF) between January 2019 and January 2024. A CHB diagnosis was defined as the presence of serum hepatitis B surface antigen (HBsAg), hepatitis B e antigen (HBeAg), or detectable serum hepatitis B virus (HBV) DNA for more than six months. Clinical data were retrieved from the hospital’s electronic medical records using a unified data framework, which encompassed serial measurements of serum ALT and HBV DNA levels throughout the follow-up period.

**Figure 1:**
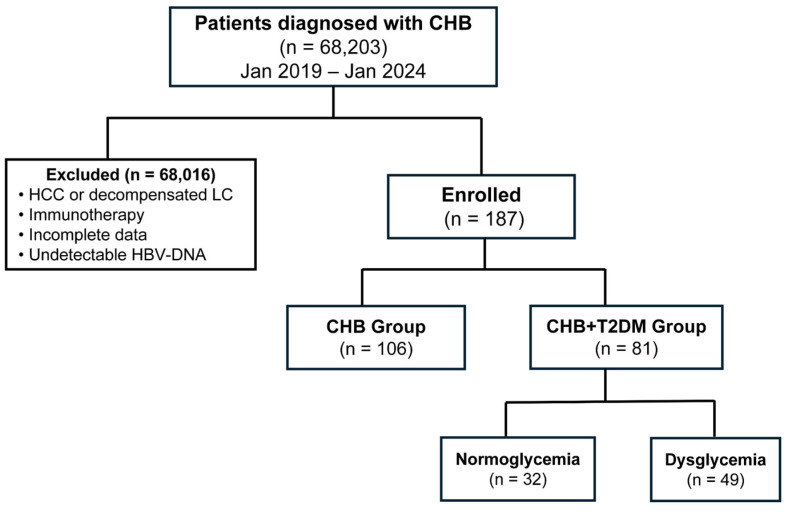
Patient flowchart. CHB, chronic hepatitis B; T2DM, type 2 diabetes mellitus; HCC, hepatocellular carcinoma; LC, liver cirrhosis; HBV, hepatitis B virus.

The exclusion criteria were as follows: (1) a diagnosis of hepatocellular carcinoma (HCC) or death within six months after treatment initiation; (2) concomitant use of immunomodulators (e.g., interferon and corticosteroids); (3) coinfection with hepatitis C virus (HCV), hepatitis D virus (HDV), or human immunodeficiency virus (HIV); or (4) insufficient clinical or laboratory data. The final enrollment of participants is shown in Figure 1.

This study was conducted in accordance with the ethical principles of the Declaration of Helsinki (1975 revision) and was approved by the Ethics Committee of the Second Affiliated Hospital of Chongqing Medical University (Approval No. 2025[266]).

### 2.2. Assessment and Definition

A biochemical response (BR) was defined as the normalization of alanine aminotransferase (ALT) levels. The upper limit of normal (ULN) was set according to the 2018 AASLD criteria at 35 IU/L for males and 25 IU/L for females [25]. The lower limit of detection (LLOD) for HBV DNA evolved from <100 IU/mL (earlier in the study period) to <20 IU/mL (later in the study period). A virological response (VR) was defined as achieving an undetectable hepatitis B virus (HBV) DNA level (below 100 IU/mL). A complete virological response (CVR) was defined as a serum HBV DNA level below 20 IU/mL at week 48 of treatment. The CVR (<20 IU/mL) was used as the primary endpoint for comparative effectiveness. This stringent threshold reflects contemporary standards of care and was chosen to specifically assess the depth of viral suppression. The VR (<100 IU/mL) was analyzed as a secondary endpoint because it was the uniformly applicable standard across the entire study period, ensuring a fair historical comparison. 

T2DM was identified based on the patient’s documented medical history. We comprehensively assessed glycemic control over the follow-up period and adjusted for potential bias due to irregular measurement intervals using the time-weighted average glycated hemoglobin (HbA1c) as a summary metric. This value was calculated as the mean of all HbA1c measurements during follow-up, weighted by the time interval between consecutive measurements.

Based on the glycemic control status, patients with T2DM were categorized into two groups: the dysglycemia group and the normoglycemia group. Dysglycemia was defined as meeting any of the following criteria: (1) a time-weighted average HbA1c level ≥ 7.0%, or (2) a fasting blood glucose level ≥ 7.0 mmol/L. Normoglycemia was defined as not meeting either of the above criteria. In cases where HbA1c and fasting glucose criteria yielded discordant classifications, the HbA1c-based criterion was used for the final determination.

Fatty liver disease and liver cirrhosis were defined based on prior medical diagnoses.

### 2.3. Statistical Analysis

Continuous variables are presented as means ± standard deviations or medians (interquartile ranges), depending on the distribution. Group comparisons were performed with Student’s *t*-test for normally distributed data, the Mann–Whitney U test for non-normally distributed data between two groups, or the Kruskal–Wallis H test for three or more groups. Variables with *p* < 0.1 in the univariate analysis were included in a multivariate binary logistic regression model to identify factors independently associated with achieving a complete virological response (CVR). The results are reported as odds ratios (ORs) with 95% confidence intervals (CIs). A two-tailed *p* < 0.05 was considered statistically significant. We evaluated whether the effect of T2DM was modified by the HBeAg status by including an interaction term (T2DM × HBeAg status) in the model. All analyses and plotting were conducted using SPSS (version 26.0.0, Armonk, NY, USA. IBM Corp) and GraphPad Prism (version 8.0.1, GraphPad Software Inc., La Jolla, CA, USA).

## 3. Results

### 3.1. Patients’ Baseline Characteristics

A total of 187 treatment-naïve patients were included in the final analysis, comprising 106 patients with chronic hepatitis B (CHB) and 81 patients with concurrent CHB and type 2 diabetes mellitus (CHB + T2DM) (Figure 1). Their baseline characteristics are summarized in Table 1. Notably, patients in the CHB + T2DM group were significantly older (median age: 52.0 vs. 41.5 years, *p* = 0.000), and a tendency toward a lower proportion with an HBeAg-positive status was observed (30.4% vs. 44.3%, *p* = 0.053). No significant intergroup differences were detected in baseline HBV DNA levels, AST levels, ALT levels, lipid profiles, or complete blood count parameters.

### 3.2. Virological Responses in the CHB and CHB + T2DM Group

**Figure 2:**
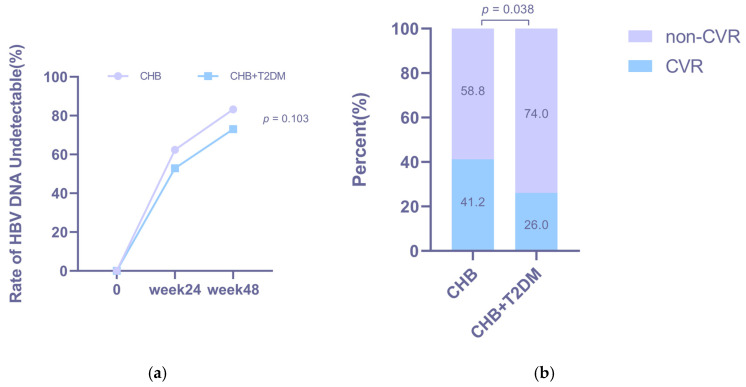

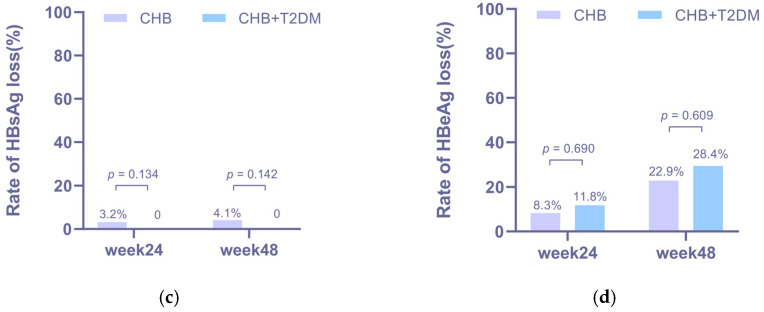
Overall antiviral response in CHB group and CHB + T2DM group after NAs treatment. Comparison of HBV DNA undetectable rates (**a**), CVR rates (**b**), HBsAg loss rates (**c**) and HBeAg loss rates (**d**) between the two groups.

Virological response rates increased over time in both groups (Figure 2a). Although the CHB + T2DM group showed a consistently lower trend than the CHB group, the differences did not reach statistical significance at either week 24 (52.9% vs. 62.4%, *p* = 0.241) or week 48 (73.0% vs. 83.2%, *p* = 0.103). In contrast, the rate of achieving a complete virological response (CVR) at week 48 was significantly lower in the CHB + T2DM group than in the CHB group (26.0% vs. 41.2%, *p* = 0.038; Figure 2b).

The HBsAg loss rates of the CHB group were 3.2% and 4.1% at week 24 and week 48, respectively (Figure 2c). Notably, no patient in the CHB + T2DM group achieved HBsAg loss during the study period. No significant difference in the HBeAg loss rate was observed between the two groups at week 24 (*p* = 0.690, Figure 2d) or week 48 (*p* = 0.609, Figure 2d).

The results of the univariate binary logistic regression analysis of factors associated with a CVR are presented in Table 2. Type 2 diabetes mellitus (T2DM) (*OR:* 0.503, 95% *CI:* 0.261–0.968, *p* = 0.040), a higher baseline hepatitis B virus (HBV) DNA level (*OR* 0.805, 95% *CI:* 0.667–0.971, *p* = 0.023), and hepatitis B e antigen (HBeAg) positivity (*OR* 0.283, 95% *CI:* 0.136–0.588, *p* = 0.001) were significant negative predictors of achieving a CVR. Conversely, adequate glycemic control (defined as a time-weighted average HbA1c < 7.0%) showed a positive association with the CVR. In the subsequent multivariate analysis, both T2DM (adjusted *OR [aOR] =* 0.406, 95% *CI:* 0.174–0.946, *p* = 0.037) and HBeAg positivity (*aOR* 0.281, 95% *CI:* 0.101–0.785, *p* = 0.015) remained independent negative predictors of a CVR. The interaction between T2DM and the HBeAg status was tested and was non-significant (*p* = 0.613), indicating that the association of T2DM with CVR was consistent across HBeAg-positive and HBeAg-negative subgroups.

### 3.3. Biochemical Responses in the CHB and CHB + T2DM Groups

**Figure 3:**
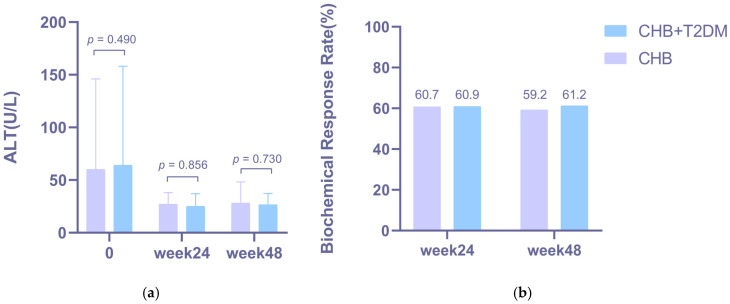
Overall ALT levels and Biochemical Response (BR) at Weeks 24 and 48 in CHB group and CHB + T2DM group after NA treatment. (**a**) Comparison of ALT levels between the CHB and CHB + T2DM groups. (**b**) Comparison of BR rates between the two groups. Error bars represent 95% confidence intervals.

After antiviral treatment, ALT levels decreased significantly in both the CHB and CHB + T2DM groups from week 0 to week 48 (*p =* 0.000, Figure 3a). Furthermore, the BR rate in patients from the CHB group was 59.2% (29/49) at week 48 (Figure 3b) and was 61.2% (30/49) in patients from the CHB + T2DM group at week 48 (Figure 3b). The ALT levels (*p* = 0.730) and BR rates (*p* = 0.836) at week 48 did not show significant differences between the two groups.

### 3.4. Subgroup Analysis of the Virological Response in CHB Patients Treated with Various NAs

We conducted a subgroup analysis to compare virological responses between the CHB and CHB + T2DM groups stratified by antiviral regimens (ETV vs. tenofovir-based therapy). The baseline characteristics of patients receiving ETV and tenofovir-based regimens are presented in Table A1 and Table A2, respectively.

**Figure 4:**
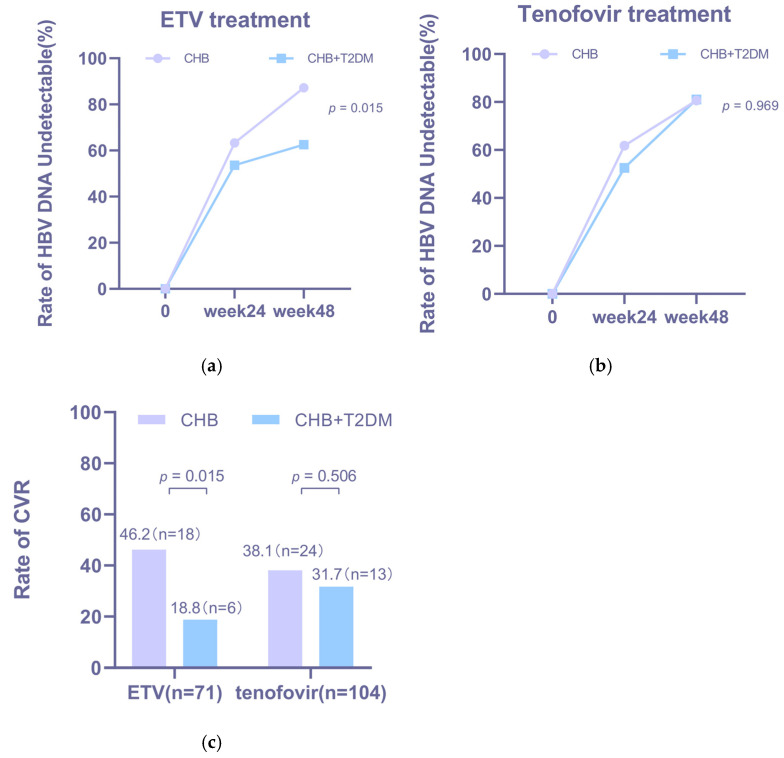
Virological Response Rates at Week 24 and Week 48 Stratified by Antiviral Agents and Presence of Type 2 Diabetes Mellitus. (**a**) Comparison of HBV DNA undetectable rates between the CHB and CHB + T2DM groups under ETV treatment. (**b**) Comparison of HBV DNA undetectable rates between the two groups under tenofovir treatment. (**c**) Comparison of CVR rates between the two groups of patients receiving different therapies.

Virological response rates increased over time in both groups across antiviral treatments (Figure 4a,b). Notably, a significantly lower CVR rate was observed in the CHB + T2DM group than in the CHB group during ETV therapy (18.8% vs. 46.2%, *p* = 0.015; Figure 4c).

**Figure 5:**
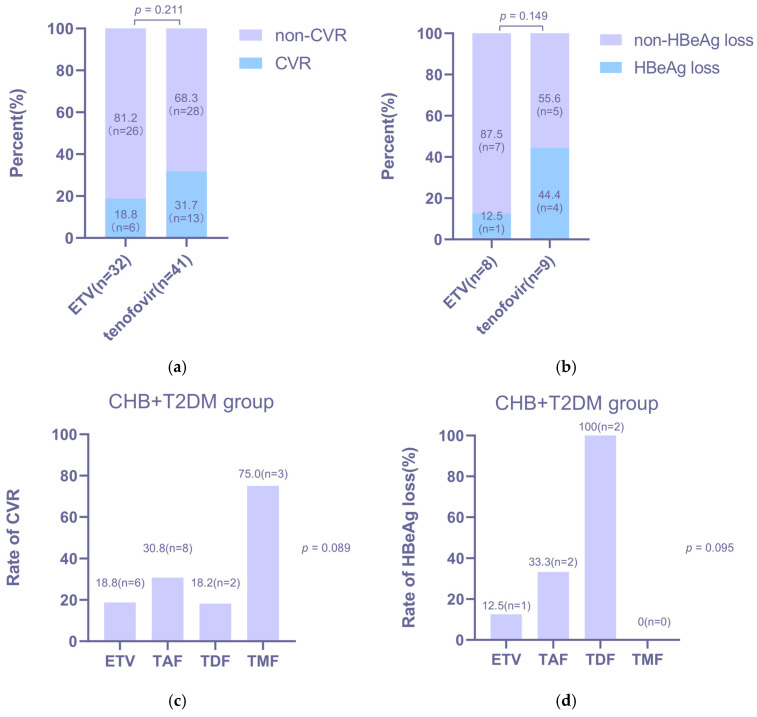
Effects of different drugs on the antiviral efficacy in CHB + T2DM groups. The effects of ETV and tenofovir on (**a**) CVR rates and (**b**) HBeAg loss rates after 48 weeks of treatment. The effects of TAF, TDF and TMF on (**c**) CVR rates and (**d**) HBeAg loss rates after 48 weeks of treatment.

To identify optimal treatment strategies for CHB patients with T2DM, we performed a within-group analysis of the CHB + T2DM cohort, comparing outcomes between patients receiving ETV and those receiving tenofovir-based regimens. The two treatment subgroups were well-matched at baseline in terms of age, sex, HBV DNA levels, and HBeAg status (Table A3). As shown in Figure 5, tenofovir-based therapy was associated with numerically higher, though not statistically significant, rates of both CVR (31.7% vs. 18.8%, *p* = 0.211; Figure 5a) and HBeAg loss (44.4% vs. 12.5%, *p* = 0.149; Figure 5b) at week 48 compared to ETV therapy. Furthermore, an exploratory analysis comparing different tenofovir formulations within the CHB + T2DM cohort suggested a trend toward superior efficacy with TDF over TAF and TMF in terms of both CVR and HBeAg loss (Figure 5c,d).

### 3.5. Association Between Glycemic Control and the Virological Response

We investigated the effect of glycemic control on antiviral efficacy by stratifying the CHB + T2DM cohort by the glycemic status (Table 3). Baseline characteristics and treatment responses for the normoglycemia (*n* = 32) and dysglycemia (*n* = 49) groups are detailed in Table 3. The two groups were well-balanced in terms of age; sex; baseline ALT, AST, and HBV DNA levels; liver stiffness measurements (LSMs); and the prevalence of cirrhosis and fatty liver, but differed significantly in the proportion of HBeAg-positive patients (12.9% vs. 41.7%, *p* = 0.007). We also conducted a systematic analysis of diabetes management strategies used for patients in the CHB + T2DM group and found no significant differences between the two groups in the use of non-insulin therapy, insulin therapy, or combination therapy (*p* = 0.108, Table 3).

Patients in the normoglycemia group achieved a significantly higher CVR rate than those in the comparison group (38.7% vs. 16.7%, *p* = 0.034; Table 3). Notably, no patients in either subgroup achieved HBsAg loss within the 48-week follow-up period. The HBeAg loss rates at weeks 24 (25.0% vs. 7.7%, *p* = 0.347) and 48 (33.3% vs. 28.6%, *p* = 0.870) were comparable, with no statistically significant intergroup differences. A subgroup analysis based on the antiviral regimen (Table 3) showed that, regardless of whether patients received ETV or tenofovir-based regimens, the normoglycemia group consistently achieved higher numerical CVR rates than the dysglycemia group (ETV: 30.0% vs. 13.6%, *p* = 0.272; tenofovir-based regimens: 42.9% vs. 20.0%, *p* = 0.116), although these differences were not statistically significant.

Given that the diabetes duration is a known modifier of liver-related outcomes [24], we further analyzed its effect on treatment efficacy. Patients newly diagnosed with diabetes at enrollment showed a trend toward higher CVR rates. Notably, they achieved a significantly higher rate of HBeAg loss at week 48 than patients with pre-existing diabetes (57.1% vs. 10.0%, *p* = 0.036; Table A4).

## 4. Discussion

In this cohort, T2DM was identified as an independent risk factor for impaired antiviral efficacy, particularly in patients receiving ETV. Optimal glycemic control (HbA1c < 7%) was strongly associated with superior virological responses. Interestingly, while T2DM comorbidity did not appear to hinder the biochemical response, it might be associated with impaired serological clearance, as evidenced by the numerically lower rates of HBsAg and HBeAg loss observed in the CHB + T2DM group than in the CHB group. Notably, our study was not powered to assess differences in serological endpoints (HBsAg/HBeAg loss) due to the extremely low absolute event rates.

While the rates of achieving the standard virological response (VR, HBV DNA < 100 IU/mL) were comparable between patients with and without diabetes, a significant difference emerged when applying the more sensitive criterion of a complete virological response (CVR, HBV DNA < 20 IU/mL). This result suggests that diabetes may primarily impair the ability to achieve deep virological suppression, a finding with implications for treatment monitoring in the era of high-sensitivity assays.

The exact biological mechanism underlying the suboptimal CVR rate in patients with T2DM remains unclear; however, impaired immune cell function may be an important contributing factor. Prior research has demonstrated that HBV infection can induce T-cell exhaustion, disrupt innate immune responses, and amplify immunosuppressive networks, ultimately leading to a dysfunctional yet persistent immune state [26,27]. Furthermore, the chronic low-grade inflammation and metabolic disturbances characteristic of T2DM are known to adversely modulate both systemic and hepatic immune microenvironments, potentially further compromising immune effector functions [28]. Consistent with this immunological perspective, our findings showed numerically lower rates of HBsAg and HBeAg loss in the CHB + T2DM group than in the CHB-only group. The observed numerical trend, albeit statistically non-significant, aligns with a conceptual premise worthy of future examination that T2DM comorbidity might partially attenuate serological clearance during therapy. We must emphasize that our study was not designed to evaluate this endpoint and lacked the statistical power to do so.

Another observation from our exploratory analyses was a differential pattern of response in patients with T2DM after stratification based on the antiviral regimen. Specifically, the negative impact of T2DM on the virological response appeared more pronounced in patients receiving entecavir (ETV) than in those receiving tenofovir-based regimens (TDF/TAF/TMF). Furthermore, within the CHB + T2DM cohort, preliminary comparisons suggested that TDF was associated with a trend toward better antiviral outcomes relative to ETV, TAF, and TMF; however, these regimen-specific subgroup analyses were statistically underpowered due to limited sample sizes and should be considered hypothesis-generating. Previous studies have suggested that replacing TAF with TDF may be a clinically reasonable consideration for certain patients, particularly for women who have gained weight and those with glucose or lipid disorders [29]. Our exploratory findings are consistent with this rationale and suggest the hypothesis that for CHB patients with comorbid T2DM, TDF might represent a preferable therapeutic option. Nevertheless, this observation requires cautious interpretation and must be confirmed in future, large-scale, prospective, and ideally randomized studies involving multi-center cohorts before any clinical recommendation can be made.

Lastly, our analysis indicated that sustained maintenance of normoglycemia appears to be associated with the achievement of a complete virological response (CVR). This observation aligns with and extends prior research linking glycemic control to long-term liver outcomes in comorbid patients. A prospective cohort study of 2330 patients identified factors reflecting the glycemic burden, including the diabetes duration, mean HbA1c level, time to target HbA1c level, and liver stiffness, as independent predictors of hepatocellular carcinoma (HCC) and fibrosis progression over three years in CHB patients with diabetes [24]. Similarly, a large retrospective analysis (*n* = 4568) indicated that among CHB patients who had achieved HBsAg seroclearance, comorbid diabetes was associated with an increased HCC risk, which was mitigated by maintaining an HbA1c level below 7% [30]. A body of evidence further confirms that poor glycemic control exacerbates the risk of adverse hepatic outcomes in this population [31,32,33]. Building on the established premise that sustained virological suppression can reverse liver fibrosis and cirrhosis [16], our findings introduce a plausible mechanistic link: maintaining optimal glycemic control (HbA1c < 7%) may increase antiviral efficacy, thereby promoting HBV DNA clearance and achieving the sustained virological suppression necessary to attenuate liver disease progression. Furthermore, our analysis revealed that a longer duration of diabetes was associated with a trend toward poorer antiviral efficacy, which was particularly reflected in lower HBeAg loss rates. This result suggests that for patients diagnosed with both CHB and T2DM, earlier intervention—potentially through the timely initiation of antiviral therapy—might be crucial to maximizing treatment response and, consequently, reducing the long-term risk of adverse liver outcomes.

However, the potential for reverse causality inherent in the observational design of this study must be acknowledged. The observed association between normoglycemia and improved virological outcomes could also be explained by the possibility that patients achieving a CVR experience better overall health, which in turn facilitates glycemic control. As such, our findings are hypothesis-generating and do not imply a definitive causal relationship. Prospective, randomized controlled trials are needed to determine whether intensive glycemic management directly increases antiviral efficacy in this patient population.

The strength of our study is that we assessed a cohort of CHB patients with diabetes with detailed continuous laboratory parameters and drug information, which is helpful to analyze the effects of diabetes and glycemic control on antiviral efficacy. We also adopted strict exclusion criteria to minimize bias as much as possible. However, several limitations should be acknowledged. First, like other retrospective studies, cases of missing data and irregular laboratory measurement intervals existed, resulting in a small sample size of patients who were ultimately included. Second, while glycemic monitoring was not protocolized, we employed the time-weighted average HbA1c level to adjust for irregular testing intervals, mitigating some related bias. Third, quantitative HBsAg data were unavailable for most patients due to assay limitations, restricting our serological analysis to a qualitative (positive/negative) assessment. Future large-scale, prospective studies should employ standardized, high-sensitivity assays to ensure complete and comparable qHBsAg datasets. Fourth, although we adjusted for key confounders, including the HBeAg status, and formally tested for an interaction, we cannot completely rule out residual confounding or more complex effect modifications by unmeasured factors. Fifth, although this study controlled for differences in healthcare-seeking behavior by rigorously assessing and documenting regular follow-up, differences in patients’ daily self-management (e.g., medication adherence) remain a potential source of residual confounding. Sixth, while we acknowledge that HBV DNA assays may have evolved during the study period, we did not conduct sensitivity analyses or restrict the analysis to later years to mitigate potential misclassification bias. Therefore, the possibility of bias due to assay changes cannot be entirely ruled out. Finally, the relatively small sample size and limited follow-up duration precluded a meaningful analysis of the association between antiviral efficacy and long-term clinical liver outcomes, such as fibrosis progression or HCC. Future prospective studies with larger, multicenter cohorts and extended follow-up are warranted to validate our findings and further elucidate the relationship between glycemic control, the virological response, and hard clinical endpoints in this comorbid population.

## 5. Conclusions

Our retrospective cohort study demonstrates that comorbid T2DM is an independent risk factor for suboptimal antiviral efficacy, specifically a lower CVR rate, in treatment-naïve patients with chronic hepatitis B. Importantly, achieving and maintaining adequate glycemic control (time-weighted average HbA1c < 7.0%) is associated with significantly improved virological outcomes. Furthermore, our regimen-specific analyses suggest that TDF may be preferable to ETV for this comorbid population. These findings underscore important clinical implications for the management of CHB patients with T2DM. Proactive screening for diabetes, its early diagnosis, and stringent glycemic control should be integral components of comprehensive care, as they may potentiate the antiviral treatment response. This integrated approach holds promise for achieving sustained virological suppression, which is fundamental to halting disease progression and reducing the long-term risk of adverse hepatic outcomes.

## Figures and Tables

**Table 1:** Baseline characteristics of all 187 patients.

Variable	Total (*n* = 187)	CHB (*n* = 106)	CHB + T2DM (*n* = 81)	*p* Value
Demographics				
Age, years	48.0 (36.0–57.0)	41.5 (31.0–53.5)	52.0 (45.0–60.0)	0.000
Male sex, *n* (%)	117 (62.6%)	71 (60.7%)	46 (56.8%)	0.154
Metabolic parameters				
HDL-C, mmol/L	1.20 (1.03–1.39)	1.21 (0.95–1.30)	1.20 (1.06–1.43)	0.390
LDL-C, mmol/L	2.45 (1.74–2.95)	2.68 (2.31–3.05)	2.29 (1.71–2.94)	0.388
Total cholesterol, mmol/L	4.47 (3.71–5.07)	4.65 (4.25–5.00)	4.38 (3.39–5.09)	0.447
Triglyceride, mmol/L	1.19 (0.91–1.65)	1.19 (0.94–1.52)	1.22 (0.90–1.71)	0.995
Fasting glucose, mmol/L	7.23 (6.14–9.55)	-	7.23 (6.14–9.55)	-
HbA1c, %	7.40 (6.50–8.80)	-	7.40 (6.50–8.80)	-
Liver function				
ALT, U/L	63.00 (35.75–149.25)	60.00 (33.00–146.00)	64.00 (36.00–158.00)	0.490
AST, U/L	48.00 (30.00–98.25)	46.00 (29.00–92.00)	49.00 (31.00–149.50)	0.253
Albumin, g/L	43.90 (39.35–46.30)	45.50 (42.18–49.41)	41.60 (36.70–45.00)	0.000
Total bilirubin, μmol/L	14.30 (10.80–21.40)	14.35 (11.65–22.14)	14.30 (10.30–20.00)	0.399
Hematological parameters				
RBC, ×10^12^/L	4.81 (4.40–5.16)	4.90 (4.49–5.24)	4.73 (4.23–5.02)	0.061
Hemoglobin, g/L	145.0 (131.8–160.0)	150.0 (133.3–160.0)	142.5 (128.0–158.0)	0.069
WBC, ×10^9^/L	5.42 (4.43–6.49)	5.37 (4.42–6.33)	5.48 (4.47–7.02)	0.361
Platelets, ×10^9^/L	164.0 (118.5–219.0)	179.0 (125.3–219.0)	160.0 (106.0–210.0)	0.134
Virological				
HBV DNA, log_10_ IU/mL	5.64 (4.14–7.06)	5.49 (4.01–7.36)	5.79 (4.79–6.51)	0.642
HBsAg positive, *n* (%)	186 (100.0%)	106 (100.0%)	80 (100.0%)	-
HBsAg, log_10_ IU/mL	3.25 (2.03–3.70)	3.41 (2.03–3.71)	3.18 (2.02–3.61)	0.537
HBeAg positive, *n* (%)	71 (38.4%)	47 (44.3%)	24 (30.4%)	0.053
Antiviral agents, *n* (%)				
ETV	74 (39.6%)	40 (37.7%)	34 (42.0%)	0.550
TAF	65 (34.8%)	34 (32.1%)	31 (38.3%)	-
TDF	32 (17.7%)	24 (22.6%)	11 (13.6%)	-
TMF	13 (7.0%)	8 (7.5%)	5 (6.2%)	-

Abbreviations: CHB, chronic hepatitis B; T2DM, type 2 diabetes mellitus; HDL-C, high-density lipoprotein cholesterol; LDL-C, low-density lipoprotein cholesterol; ALT, alanine aminotransferase; AST, aspartate aminotransferase; RBC, red blood cell; WBC, white blood cell; HBV, hepatitis B virus; HBeAg, hepatitis B e antigen; HBsAg, hepatitis B surface antigen; ETV, Entecavir; TAF, tenofovir alafenamide; TDF, tenofovir disoproxil fumarate; TMF, tenofovir amibufenamide.

**Table 2:** Binary logistic regression for factors associated with CVR at week 48.

Variable	Univariate Analysis			Multivariate Analysis		
	OR	95% CI	*p* Value	OR	95%CI	*p* Value
T2DM	0.503	0.261–0.968	0.040	0.406	0.174–0.946	0.037
Time-weighted mean HbA1c < 7%	3.158	1.065–9.361	0.038	NA	NA	NA
Age	1.017	0.993–1.041	0.161			
Male	0.566	0.299–1.702	0.081	0.558	0.271–1.149	0.113
HDL-C	1.055	0.869–1.281	0.588			
LDL-C	1.077	0.861–1.347	0.515			
Total cholesterol	0.753	0.498–1.139	0.179			
Triglyceride	0.899	0.604–1.339	0.602			
ALT	1.000	0.999–1.001	0.992			
AST	1.000	0.998–1.001	0.687			
Albumin	1.005	0.993–1.120	0.084	1.041	0.972–1.115	0.253
Total bilirubin	0.999	0.993–1.006	0.830			
RBC	0.849	0.499–1.442	0.544			
Hemoglobin	0.996	0.979–1.103	0.655			
WBC	1.071	0.934–1.229	0.326			
Platelets	1.002	0.997–1.006	0.523			
HBV DNA	0.805	0.667–0.971	0.023	0.950	0.753–1.199	0.668
HBsAg	0.828	0.506–1.353	0.451			
HBeAg positive	0.283	0.136–0.588	0.001	0.281	0.101–0.785	0.015
T2DM × HBeAg status	0.082	0.011–0.625	0.016	0.543	0.051–5.774	0.613
ETV	-	-	0.762			
TAF	0.613	0.170–2.214	0.455			
TDF	0.714	0.195–2.615	0.610			
TMF	0.500	0.124–2.022	0.331			

Abbreviations: T2DM, type 2 diabetes mellitus; HDL-C, high-density lipoprotein cholesterol; LDL-C, low-density lipoprotein cholesterol; ALT, alanine aminotransferase; AST, aspartate aminotransferase; RBC, red blood cell; WBC, white blood cell; HBV, hepatitis B virus; HBeAg, hepatitis B e antigen; HBsAg, hepatitis B surface antigen; ETV, Entecavir; TAF, tenofovir alafenamide; TDF, tenofovir disoproxil fumarate; TMF, tenofovir amibufenamide. NA: The HbA1c parameter was not included in the multivariate model because this parameter was unavailable for patients without diabetes. Its inclusion would have necessitated the exclusion of all non-diabetic patients from the regression, thereby preventing an analysis of the entire cohort.

**Table 3:** Baseline characteristics and antiviral response of CHB + T2DM group stratified by glycemic control status.

Variable	Total (*n* = 81)	Normoglycemia (HbA1c < 7%) (*n* = 32)	Dysglycemia (HbA1c ≥ 7%) (*n* = 49)	*p* Value
General				
Mean age (years)	52.83 ± 12.12	52.38 ± 12.53	53.12 ± 11.96	0.788
Sex, male (%)	46 (56.8%)	17 (53.1%)	29 (59.2%)	0.590
Metabolic parameters				
HDL-cholesterol (mmol/L)	1.20 (1.06–1.43)	1.20 (1.06–1.40)	1.20 (0.98–1.53)	0.859
LDL-cholesterol (mmol/L)	2.29 (1.71–2.94)	2.26 (1.69–2.75)	2.40 (1.72–3.41)	0.373
Total cholesterol (mmol/L)	4.38 (3.39–5.09)	4.33 (3.44–4.70)	4.74 (3.37–5.19)	0.545
Triglyceride (mmol/L)	1.22 (0.90–1.71)	1.18 (0.89–1.59)	1.25 (0.90–2.16)	0.66
Fasting glucose (mmol/L)	7.23 (6.14–9.55)	6.35 (5.38–7.69)	7.94 (6.65–12.70)	0.002
Time-weighted mean HbA1c, %	7.20 (6.37–8.50)	6.35 (5.80–6.68)	8.32 (7.63–10.40)	0.000
Liver function				
ALT (U/L)	265.83 ± 529.75	304.78 ± 549.07	240.39 ± 520.92	0.596
AST (U/L)	171.85 ± 319.35	191.72 ± 333.51	158.88 ± 312.57	0.654
Albumin (g/L)	41.60 (36.70–45.10)	42.75 (38.10–45.45)	40.40 (33.70–44.20)	0.171
Total bilirubin (umol/L)	14.30 (10.30–20.60)	13.00 (9.15–20.28)	14.80 (11.90–22.30)	0.231
LSM (kPa)	12.90 (6.90–26.80)	10.00 (7.00–16.45)	13.10 (6.90–26.90)	0.424
Fatty liver disease (*n*,%)	18 (22.2%)	6 (18.8%)	12 (24.5%)	0.544
Liver cirrhosis (*n*,%)	29 (35.8%)	8 (25.0%)	21 (42.9%)	0.101
Diabetes treatment strategy				
Untreated, *n (*%)	13 (16.0%)	4 (12.5%)	9 (18.4%)	0.108
Non-insulin therapies, *n (*%)	43 (53.1%)	20 (62.5%)	23 (46.9%)	-
Insulin therapy, *n (*%)	14 (17.3%)	2 (6.3%)	12 (24.5%)	-
Combination therapy, *n (*%)	11 (13.6%)	6 (18.8%)	5 (10.2%)	-
Virological				
HBV DNA (log10 IU/mL)	5.67 ± 1.50	5.79 ± 1.38	5.58 ± 1.59	0.539
HBsAg, positive (%)	80 (100%)	32 (100%)	48 (100%)	-
HBsAg (log10 IU/mL)	2.89 ± 0.84	2.84 ± 0.68	2.91 ± 0.92	0.876
HBeAg, positive (%)	24 (30.4%)	4 (12.9%)	20 (41.7%)	0.007
HBsAg loss (%)				
Week 24	0	0	0	-
Week 48	0	0	0	-
HBeAg loss (%)				
Week 24	2/17 (11.8%)	1/4 (25.0%)	1/13 (7.7%)	0.347
Week 48	5/17 (29.4%)	1/3 (33.3%)	4/14 (28.6)	0.870
Complete virological response (%)	19/73 (26.0%)	12/31 (38.7%)	7/42 (16.7%)	0.034
Antiviral treatment strategy				
ETV treated	*n* = 34	*n* = 10	*n* = 24	
Complete Virological Response (%)	6/32 (18.8%)	3/10 (30.0%)	3/22 (13.6%)	0.272
Tenofovir treated	*n* = 47	*n* = 22	*n* = 25	
Complete Virological Response (%)	13/41 (31.7%)	9/21 (42.9%)	4/20 (20.0%)	0.116

Abbreviations: ALT, alanine aminotransferase; AST, aspartate aminotransferase; LSM, Liver stiffness measurement; HBV, hepatitis B virus; HBeAg, hepatitis B e antigen; HBsAg, hepatitis B surface antigen; ETV, Entecavir.

## Data Availability

The data that support the findings of this study are available from the corresponding authors upon reasonable request.

## Abbreviations

The following abbreviations are used in this manuscript:

CHB	Chronic hepatitis B
T2DM	Type 2 diabetes mellitus
NAs	Nucleos(t)ide analogues
CVR	Complete virological response
HCC	Hepatocellular carcinoma
LC	liver cirrhosis
ALT	Alanine aminotransferase
AST	Aspartate aminotransferase
HBV	Hepatitis B virus
HBeAg	Hepatitis B e antigen
HBsAg	Hepatitis B surface antigen
cccDNA	Covalently closed circular DNA
OR	Odds ratio
CI	Confidence interval
aHR	Adjusted hazard ratio
aOR	Adjusted odds ratio
ETV	Entecavir
TAF	Tenofovir alafenamide
TDF	Tenofovir disoproxil fumarate
TMF	Tenofovir amibufenamide
RBC	Red blood cell
WBC	White blood cell
HCV	Hepatitis C virus
HDV	Hepatitis D virus
HIV	Human immunodeficiency virus
AASLD	American Association for the Study of Liver Diseases
BR	Biochemical response
VR	Virological response
ULN	Upper limit of normal
HbA1c	Hemoglobin A1c
IQR	Interquartile range
HDL-C	High-density lipoprotein cholesterol
LDL-C	Low-density lipoprotein cholesterol

## Appendix A

**Table A1:** Baseline characteristics of patients with ETV treatment.

Variable	Total (*n* = 74)	CHB (*n* = 40)	CHB + T2DM (*n* = 34)	*p* Value
Demographics				
Age, years	48.62 ± 13.26	44.93 ± 12.86	52.97 ± 12.56	0.008
Male sex, *n* (%)	44 (59.5%)	23 (57.5%)	21 (61.8%)	0.710
Metabolic parameters				
HDL-C, mmol/L	1.14 (0.97–1.32)	1.19 (0.93–1.33)	1.12 (1.00–1.32)	0.919
LDL-C, mmol/L	2.68 (1.79–3.45)	2.79 (2.37–3.27)	2.36 (1.66–3.71)	0.473
Total cholesterol, mmol/L	4.61 ± 1.37	5.12 ± 1.44	4.38 ± 1.30	0.159
Triglyceride, mmol/L	1.20 (0.85–1.76)	1.14 (0.84–1.61)	1.36 (0.81–1.88)	0.735
Fasting glucose, mmol/L	7.09 (6.13–8.99)	-	7.09 (6.13–8.99)	-
HbA1c, %	7.40 (6.90–10.60)	-	7.40 (6.90–10.60)	-
Liver function				
ALT, U/L	64.00 (32.75–144.50)	60.50 (32.00–196.00)	65.50 (35.25–97.50)	0.983
AST, U/L	51.00 (28.00–101.50)	50.00 (29.00–106.50)	52.00 (27.25–90.25)	0.939
Albumin, g/L	42.60 (39.50–46.15)	45.40 (41.30–46.90)	41.55 (36.43–43.53)	0.001
Total bilirubin, μmol/L	14.30 (11.65–22.35)	14.35 (11.70–28.40)	14.10 (11.25–16.65)	0.444
Hematological parameters				
RBC, ×10^12^/L	4.70 ± 0.56	4.74 ± 0.53	4.65 ± 0.59	0.519
Hemoglobin, g/L	141.07 ± 19.60	143.57 ± 19.27	138.27 ± 19.87	0.262
WBC, ×10^9^/L	5.44 (4.48–6.37)	5.29 (4.28–6.00)	5.73 (4.51–6.97)	0.239
Platelets, ×10^9^/L	168.04 ± 71.68	176.05 ± 67.10	159.06 ± 76.54	0.326
Virological				
HBV DNA, log_10_ IU/mL	5.25 (4.00–6.50)	4.82 (3.66–6.77)	5.58 (4.68–6.41)	0.160
HBsAg positive, *n* (%)	74 (100.0%)	40 (100.0%)	34 (100%)	-
HBsAg, log_10_ IU/mL	3.25 (1.78–3.47)	3.25 (1.73–3.52)	3.27 (1.91–3.56)	0.779
HBeAg positive, *n* (%)	23 (31.1%)	12 (30.0%)	11 (32.4%)	0.827

Abbreviations: CHB, chronic hepatitis B; T2DM, type 2 diabetes mellitus; HDL-C, high density lipoprotein cholesterol; LDL-C, low density lipoprotein cholesterol; ALT, alanine aminotransferase; AST, aspartate aminotransferase; RBC, red blood cell; WBC, white blood cell; HBV, hepatitis B virus; HBeAg, hepatitis B e antigen; HBsAg, hepatitis B surface antigen.

**Table A2:** Baseline characteristics of patients with tenofovir treatment.

Variable	Total (*n* = 113)	CHB (*n* = 66)	CHB + T2DM (*n* = 47)	*p* Value
Demographics				
Age, years	47.00 (34.00–56.50)	38.50 (30.75–53.00)	51.00 (44.00–59.00)	0.000
Male sex, *n* (%)	73 (64.6%)	48 (72.7%)	25 (53.2%)	0.032
Metabolic parameters				
HDL-C, mmol/L	1.26 ± 0.37	1.14 ± 0.37	1.30 ± 037	0.241
LDL-C, mmol/L	2.35 (1.71–2.82)	2.56 (1.45–2.84)	2.26 (1.71–3.29)	0.844
Total cholesterol, mmol/L	4.38 (3.90–4.99)	4.48 (3.79–4.97)	4.33 (3.89–5.22)	0.844
Triglyceride, mmol/L	1.19 (0.94–1.53)	1.21 (1.09–1.55)	1.18 (0.91–1.54)	0.627
Fasting glucose, mmol/L	7.27 (6.14–10.15)	-	7.27 (6.14–10.15)	-
HbA1c, %	6.90 (6.10–8.60)	-	6.90 (6.10–8.60)	-
Liver function				
ALT, U/L	61.00 (40.00–150.00)	60.00 (39.00–141.50)	62.00 (39.00–244.00)	0.361
AST, U/L	47.00 (30.00–96.75)	45.00 (29.00–86.00)	48.00 (35.00–183.00)	0.147
Albumin, g/L	44.50 (39.23–46.40)	45.60 (43.20–47.30)	42.80 (36.55–45.80)	0.009
Total bilirubin, μmol/L	14.60 (10.48–21.18)	14.30 (11.20–21.28)	15.30 (9.85–21.40)	0.691
Hematological parameters				
RBC, ×10^12^/L	4.85 (4.41–5.23)	4.95 (4.58–5.26)	4.68 (4.15–5.16)	0.097
Hemoglobin, g/L	146.50 (132.00–161.00)	152.00 (134.00–161.00)	144.00 (130.00–161.00)	0.244
WBC, ×10^9^/L	5.42 (4.42–6.77)	5.41 (4.44–6.35)	5.43 (4.33–7.20)	0.770
Platelets, ×10^9^/L	169.22 ± 68.74	173.83 ± 66.10	162.78 ± 72.55	0.413
Virological				
HBV DNA, log_10_ IU/mL	5.85 (4.28–7.56)	5.80 (4.11–8.011)	6.08 (4.76–6.57)	0.608
HBsAg positive, *n* (%)	112 (100.0%)	66 (100.0%)	46 (100.0%)	-
HBsAg, log_10_ IU/mL	3.11 ± 1.13	3.24 ± 1.25	2.87 ± 0.86	0.375
HBeAg positive, *n* (%)	48 (43.2%)	35 (53.0%)	13 (28.9%)	0.012

Abbreviations: CHB, chronic hepatitis B; T2DM, type 2 diabetes mellitus; HDL-C, high-density lipoprotein cholesterol; LDL-C, low-density lipoprotein cholesterol; ALT, alanine aminotransferase; AST, aspartate aminotransferase; RBC, red blood cell; WBC, white blood cell; HBV, hepatitis B virus; HBeAg, hepatitis B e antigen; HBsAg, hepatitis B surface antigen.

**Table A3:** Baseline characteristics of CHB + T2DM group stratified by different antiviral treatment.

Variable	Total (*n* = 81)	ETV (*n* = 34)	Tenofovir (*n* = 47)	*p* Value
General				
Age, years	52.83 ± 12.12	52.97 ± 12.56	52.72 ± 11.92	0.928
Male sex, *n* (%)	46 (56.8%)	21 (61.8%)	25 (53.2%)	0.442
Metabolic parameters				
HDL-C, mmol/L	1.20 (1.06–1.43)	1.12 (1.00–1.32)	1.22 (1.11–1.59)	0.170
LDL-C, mmol/L	2.29 (1.71–2.94)	2.36 (1.66–3.71)	2.26 (1.71–2.78)	0.557
Total cholesterol, mmol/L	4.38 (3.39–5.09)	4.39 (3.17–5.09)	4.33 (3.89–5.22)	0.718
Triglyceride, mmol/L	1.22 (0.90–1.71)	1.36 (0.81–1.88)	1.18 (0.91–1.54)	0.845
Fasting glucose, mmol/L	7.23 (6.14–9.55)	7.09 (6.13–8.99)	7.27 (6.14–10.15)	0.675
HbA1c, %	7.40 (6.50–8.80)	7.40 (6.90–11.48)	6.90 (6.10–8.60)	0.209
Liver function				
ALT, U/L	64.00 (36.00–158.00)	65.50 (35.25–97.50)	62.00 (244.00)	0.348
AST, U/L	49.00 (31.00–149.50)	52.00 (27.25–90.25)	48.00 (35.00–183.00)	0.476
Albumin, g/L	41.60 (36.70–45.10)	41.55 (36.43–43.53)	42.80 (36.55–45.80)	0.229
Total bilirubin, μmol/L	14.30 (10.30–20.60)	14.10 (11.25–16.65)	15.30 (9.85–21.40)	0.929
Hematological parameters				
RBC, ×10^12^/L	4.67 ± 0.62	4.65 ± 0.59	4.69 ± 0.65	0.798
Hemoglobin, g/L	140.99 ± 19.53	138.27 ± 19.87	142.98 ± 19.25	0.296
WBC, ×10^9^/L	5.48 (4.47–7.02)	5.73 (4.51–6.97)	5.43 (4.33–7.20)	0.887
Platelets, ×10^9^/L	161.21 ± 73.79	159.06 ± 76.54	162.78 ± 72.55	0.828
Virological				
HBV DNA, log_10_ IU/mL	5.67 ± 1.50	5.54 ± 1.48	5.76 ± 1.53	0.507
HBsAg positive, *n* (%)	81 (100%)	34 (100%)	47 (100%)	-
HBsAg, log_10_ IU/mL	2.89 ± 0.84	2.92 ± 0.88	2.87 ± 0.86	0.906
HBeAg positive, *n* (%)	24 (30.4%)	11 (32.4%)	13 (28.9%)	0.740

Abbreviations: CHB, chronic hepatitis B; T2DM, type 2 diabetes mellitus; HDL-C, high-density lipoprotein cholesterol; LDL-C, low-density lipoprotein cholesterol; ALT, alanine aminotransferase; AST, aspartate aminotransferase; RBC, red blood cell; WBC, white blood cell; HBV, hepatitis B virus; HBeAg, hepatitis B e antigen; HBsAg, hepatitis B surface antigen.

**Table A4:** Baseline characteristics and antiviral response of CHB + T2DM group stratified by duration of T2DM.

Variable	Total (*n* = 81)	Newly Diagnosed (*n* = 28)	Pre-Existing (*n* = 53)	*p* Value
General				
Mean age (years)	52.83 ± 12.12	48.39 ± 11.04	55.17 ± 12.10	0.016
Sex, male (%)	46 (56.8%)	18 (64.3%)	28 (52.8%)	0.322
Metabolic parameters				
HDL-cholesterol (mmol/L)	1.20 (1.06–1.43)	1.16 (0.96–1.44)	1.21 (1.07–1.47)	0.686
LDL-cholesterol (mmol/L)	2.29 (1.71–2.94)	2.25 (1.73–3.71)	2.29 (1.67–2.91)	0.343
Total cholesterol (mmol/L)	4.38 (3.39–5.09)	4.25 (3.38–5.07)	4.43 (3.49–5.19)	0.947
Triglyceride (mmol/L)	1.22 (0.90–1.71)	1.36 (1.09–1.88)	1.07 (0.85–1.64)	0.275
Fasting glucose (mmol/L)	7.23 (6.14–9.55)	7.28 (6.66–9.10)	7.20 (5.93–10.04)	0.444
HbA1c, %	7.40 (6.50–8.80)	7.90 (7.20–10.80)	6.90 (6.20–8.50)	0.040
Liver function				
ALT (U/L)	64.00 (36.00–158.00)	63.50 (46.00–102.25)	69.00 (34.50–238.00)	0.921
AST (U/L)	49.00 (31.00–149.50)	51.00 (33.50–70.25)	49.00 (29.50–172.50)	0.687
Albumin (g/L)	40.75 ± 5.99	40.92 ± 5.59	40.65 ± 6.24	0.852
Total bilirubin (umol/L)	14.30 (10.30–20.60)	13.30 (10.80–18.40)	14.35 (9.93–22.98)	0.824
Virological & Treatment				
HBV DNA (log10 IU/mL)	5.67 ± 1.50	5.18 ± 1.44	5.93 ± 1.48	0.032
HBsAg, positive(%)	80 (100%)	28 (100%)	52 (100%)	-
HBsAg (log10 IU/mL)	3.18 (2.02–3.61)	3.21 (2.00–3.55)	3.18 (1.97–3.78)	0.859
HBeAg, positive(%)	24 (30.4%)	10 (35.7%)	14 (27.5%)	0.445
ETV, *n* (%)	34 (42.0%)	15 (53.6%)	19 (35.8%)	0.136
TAF, *n* (%)	31 (38.3%)	8 (28.6%)	23 (43.4%)	-
TDF, *n* (%)	11 (13.6%)	5 (17.9%)	6 (11.3%)	-
TMF, *n*(%)	5 (6.2%)	0 (0%)	5 (9.4%)	-
HBV DNA undetectable (%)				
Week 24	36/68 (52.9%)	13/24 (54.2%)	23/44 (52.3%)	0.881
Week 48	54/74 (73.0%)	19/27 (70.4%)	35/47 (74.5%)	0.702
HBsAg loss (%)				
Week 24	0	0	0	-
Week 48	0	0	0	-
HBeAg loss (%)				
Week 24	2/17 (11.8%)	2/7 (28.6%)	0/10 (0%)	0.072
Week 48	5/17 (29.4%)	4/7 (57.1%)	1/10 (10.0%)	0.036
Complete virological response	19/73 (26.0%)	7/27 (25.9%)	12/46 (26.1%)	0.988

Abbreviations: ALT, alanine aminotransferase; AST, aspartate aminotransferase; HBV, hepatitis B virus; HBeAg, hepatitis B e antigen; HBsAg, hepatitis B surface antigen; ETV, entecavir; TAF, tenofovir alafenamide; TDF, tenofovir disoproxil fumarate; TMF, tenofovir amibufenamide.
